# The Cardio-Oncology Patients—What They Know and What They Should Know

**DOI:** 10.3390/curroncol32110613

**Published:** 2025-11-02

**Authors:** Aneta Klotzka, Barbara Gawłowska, Ewelina Chawłowska

**Affiliations:** 1 1st Department of Cardiology, Poznan University of Medical Sciences, 61-848 Poznan, Poland; aneta.klotzka@usk.poznan.pl; 2 Department of Preventive Medicine, Poznan University of Medical Sciences, 60-781 Poznan, Poland; bgawlowska@ump.edu.pl

**Keywords:** cardio-oncology, health behaviours, health education, cancer patients, physicians

## Abstract

**Simple Summary:**

Cardiovascular complications have become increasingly common among cancer survivors, largely due to improved treatment outcomes and longer survival. As events like myocardial infarction and heart failure now significantly affect long-term health, assessing patients’ awareness of these risks is essential. The purpose of this study was to gather information on respondents’ knowledge of their treatment, awareness of cardiovascular risks associated with cancer therapy, and the factors associated with these outcomes. The level of awareness was related to age, education, as well as health behaviors and communication with the physician.

**Abstract:**

The growing number of patients after oncological treatment makes knowledge about potential cardiovascular complications of cancer therapy particularly important. Early recognition of symptoms enables the rapid initiation of appropriate therapy and improves outcomes. Education in this field increases awareness of the need for regular cardiology follow-up and adherence to health recommendations. It is advisable for patient education on the risk of cardiotoxicity to be included during visits with both the oncologist and the cardiologist. A self-developed questionnaire was used. It consisted of 40 questions (including 16 from the Health Behavior Scale) and 8 additional sociodemographic questions. An anonymous questionnaire was completed by 243 patients of the cardio-oncology outpatient clinic operating within the Department of Cardiology in Poland. In the survey conducted, patients were asked to define the concept of cardio-oncology; only 23.5% of respondents provided a correct answer. The highest level of awareness was observed among individuals under the age of 40 (*p* = 0.001) and of higher education levels (*p* < 0.001). Better knowledge was also noted among respondents who recalled being informed by their doctor about complications (*p* < 0.001) and among those who had undergone cardiological examinations (*p* = 0.005). The findings further revealed that respondents who recognized the importance of cardiac monitoring following therapy were significantly more likely to engage in health behaviors (*p* < 0.001). Particularly concerning was the limited communication regarding cardiovascular risks associated with cancer treatment. Only 24.3% of patients reported having been informed (or recalled being informed) by their oncologist about the potential cardiotoxic effects of anticancer drugs. Approximately one-third of respondents (32%) had not been referred for a cardiology consultation during their cancer treatment. Despite this, an overwhelming majority (95.5%) expressed the belief that a cardiologist should assess all oncology patients. These findings underscore critical deficiencies in patients’ education within the field of cardio-oncology. Health education interventions during oncological follow-up visits are needed

## 1. Introduction

Cardio-oncology is a relatively young but rapidly developing branch of medicine. It combines cardiology and oncology. Both of these fields are associated with the largest number of morbidity cases in the world, and thus the largest number of deaths. Cardio-oncology addresses the cardiac complications of oncological treatment at every stage of cancer therapy. It therefore includes in its care patients undergoing oncological treatment who develop cardiac complications [1]. Its main goal, however, is early recognition and prevention so that such cardiac complications do not occur. It also focuses on the long-term follow-up of patients who have received oncological treatment in the past and who may develop cardiac complications years later [2,3]. Finally, cardio-oncology also covers patients with pre-existing heart disease who are eligible for oncologic treatment, requiring an individualized approach and monitoring [4].

Arguably, the term “cardio-oncology” first appeared in 1996 and was solidified through the work of Dr. Daniel Lenihan and Dr. Steven Lipshultz in the United States [5]. Although the cardiovascular toxicity of anthracyclines was already noted in works from the late 20th century, the development of this branch under the name “cardio-oncology” has largely taken place over the past decade [5,6].

Cardio-oncologic complications extend beyond the duration of oncologic treatment. Chemotherapy, radiotherapy, or immunotherapy, which are widely used in oncology, can lead to adverse cardiovascular events both during and years after oncological treatment.

The frequency of cardiovascular complications in people undergoing oncologic treatment is influenced by, among other factors, the type of therapy, the patient’s age, and pre-existing comorbidities. Identifying populations at higher risk of complications is crucial [7,8,9].

The scope of cardiac complications is broad. It covers the entire spectrum of cardiology. Among them, we can mention heart failure, thromboembolic complications, myocarditis, rhythm disorders, and hypertension [10]. Although anthracyclines are still widely used, other forms of treatment, such as radiation therapy to the chest and immunotherapy, which are increasingly being integrated into treatment regimens, also carry a risk of cardiac complications [11].

Many of these cardiovascular complications could be avoided or their effects reduced with cardioprotective behaviors. Earlier diagnosis of them can be enabled by focusing on preventive examinations [12,13]. For this to happen, however, a high level of awareness of the occurrence of such complications on the part of both the patient and physicians (including oncologists, cardiologists, and primary care physicians) is essential [12]. Unfortunately, cardiac complications are the leading cause of death among cancer survivors.

Specialized cardiac oncology units are increasingly being established in cancer centers around the world to provide comprehensive care in this area [1]. Scientific societies such as the European Society of Cardiology (ESC), the American Society of Clinical Oncology (ASCO), and the European Society for Medical Oncology (ESMO) have published dedicated guidelines on cardiovascular complications and risk assessment in oncology patients [1,2]. The current 2022 European ESC guidelines are the most comprehensive document on the subject and provide specific diagnostic and therapeutic recommendations [1]. Thus, we are witnessing a growing awareness among physicians about cardio-oncologic complications. Doctors are often the main source of health education, i.e., the knowledge and skills that enable patients to take care of their health [14,15,16]. Health education is an important factor in developing patients’ health literacy.

Therefore, patient education and close cooperation between the oncologist and cardiologist are essential.

Awareness is highest among younger individuals, while patients with multiple comorbidities tend to be less familiar with the concept of cardio-oncology]. Lifestyle factors—including physical activity and a balanced diet—play a critical role not only in cardiovascular disease prevention but also in supporting cancer treatment outcomes and improving prognosis [6,12].

We surveyed to answer questions about the awareness of cardiac complications among cardio-oncology patients who were undergoing or had undergone oncologic treatment. The survey also aimed to assess whether awareness and health knowledge of the patients correlated with their health behavior. We also aimed to evaluate the role of physician-led health education in shaping a patient’s health literacy. This study will help to formulate specific educational activities aimed at cardio-oncology patients.

## 2. Materials and Methods

### 2.1. Sampling

A cross-sectional study was conducted among 243 patients attending an outpatient cardio-oncology clinic in Poznan, Poland. Participants were informed about the aim of the study while in the clinic waiting room. Subsequently they completed an anonymous questionnaire voluntarily after the visit to the office. Completing the paper-and-pencil survey took an average of 10–15 min. If necessary, patients could count on professional assistance in completing the questionnaire. They could ask questions while completing the survey and withdraw from the study at any time. Eligibility criteria included being an adult and having a confirmed diagnosis of any type of cancer. Data collection was carried out between October 2023 and April 2024. According to the statement KB–919/22 issued by the Bioethics Committee of the Poznan University of Medical Sciences (Poznan, Poland) the study did not constitute a scientific experiment.

### 2.2. Measurement

Based on a review of the literature and relevant recommendations, a structured questionnaire was developed, comprising three sections: (1) questions related to cardio-oncology, (2) questions related to health behaviors, and (3) sociodemographic items. 37 items were single-choice, and 3 were multiple-choice. The tool was reviewed by four subject-matter experts in the fields of cardiology, public health, patient education, and healthcare.

The following questions examined patients’ awareness of cardiac complications:

- What is cardio-oncology?
- In your opinion: Can chemotherapy “damage” the heart?
- In your opinion: Should the heart be monitored during chemotherapy?
- In your opinion: Should one go to a cardiologist for follow-up after cancer treatment?
- In your opinion: Should the heart be monitored after radiation therapy to the chest?
- In your opinion: Can radiation therapy to the chest “damage” the heart?
- Other questions concerned examinations and providing information by the doctor:
- Did your doctor inform you about possible cardiac complications caused by cancer drugs?
- Did your doctor inform you about possible cardiac complications caused by radiation therapy to the chest?
- Did a cardiologist examine you during cancer treatment?
- Did you have an echocardiogram. known as a cardiac echo. during your cancer treatment?

The response options “no” and “I don’t know” or “I don’t remember” were combined during the statistical process because we wanted to obtain a clear classification of respondents in terms of their knowledge. Differentiating between a lack of knowledge and an uncertainty about one’s knowledge did not result in a significant statistical difference.

To assess patients’ health behaviors, 16 items from Health Behavior Scale (HBS) were utilized [17]. This instrument evaluates five domains: diet, physical activity, harmful behaviors, individual attitudes, and preventive behaviors within the healthcare system. The specific questions in this tool are provided in the Supplementary Material (Table S1: Health Behaviour Scale). Responses were coded on a 0–3 scale, with higher scores indicating more health-promoting behavior. The maximum possible HBS score was 45 points. The instrument was designed to gather information about participants’ knowledge of their cancer treatment, awareness of associated cardiovascular risks, and engagement in both health-promoting and harmful behaviors.

### 2.3. Statistical Analysis

Statistical analyses were performed using PQStat software, v1.8.6 [18]. The Shapiro–Wilk test was used to assess the normality of the data distribution, which proved to be non-parametric. To assess relationships between variables, appropriate statistical tests were applied, including the chi^2^ test, and the Mann–Whitney U test. The Mann–Whitney U test was used to calculate the statistical difference between variables on an interval scale (health behavior score) or ordinal scale (age, education, financial situation, BMI) and nominal scale (cardio-oncology knowledge). The chi^2^ test was used to assess the statistical difference between categorical variables such as cardio-oncology knowledge and therapy type. This test was also used for questions concerning patients’ awareness of cardiac complications. Possible multiple comparisons were calculated using the Bonferroni correction. A significance threshold of *p* = 0.05 was used for all analyses

## 3. Results

### 3.1. Sample Characteristics

Table 1 presents the descriptive characteristics of the study population. The response rate was 80%. The majority of participants were women, comprising 75.7% of the total sample. The mean age of respondents was 51.2 years (SD = 9.8), and the mean Body Mass Index (BMI), calculated based on self-reported weight and height, was 27.0 (SD = 18.7). More than half of the participants (53.5%) were classified as having an elevated BMI [19]. The largest educational subgroup included individuals with secondary education (38.7%). Additionally, over half of the sample resided in large urban areas (53.5%) and were actively employed (61.7%). Only a small proportion of respondents (9.1%) rated their financial situation as good or very good. Breast cancer was the most commonly reported cancer type, accounting for 55.6% of cases. In terms of treatment, approximately 32% of patients reported receiving both chemotherapy and radiotherapy, whereas nearly 27% were unaware of the specific oncological treatment they had undergone.

### 3.2. Knowledge About Cardiac Complications

Patients’ awareness of cardiac complications associated with their cancer treatment and the relationship with various demographic variables were analyzed (Table 2). In questions concerned patients’ awareness, examinations and providing information by the doctor the response options “no” and “I don’t know” or “I don’t remember” were combined during statistical process. We wanted to obtain a clear classification of respondents in terms of their knowledge. The respondents were divided into two groups—those having knowledge and the rest—in order to examine the differences between them. As a result, we observed a statistically significant difference between these groups. Patients were asked to select the correct definition of cardio-oncology. Only 23.5% of respondents knew what cardio-oncology is. Statistical significance was identified in age, education level, BMI and type of oncological therapy received by the patient. Among patients under 40 years old, awareness of cardiac complications was the highest. This awareness declined with increasing age and increased with the level of education. Additionally, most individuals with obesity and those unaware of the type of oncological therapy they were receiving also lacked knowledge of cardio-oncology. Statistical significance in relation to financial situation was identified only in questions concerning complications after chemotherapy. Patients who declared an average financial situation were least likely to say that the heart should be monitored.

### 3.3. The Physician’s Role in Communicating Cardiac Complications

Table 3 shows a significant correlation between patient awareness and physician actions. Only 24.3% of patients were informed by their oncologist (or remember being informed) about possible cardiac complications caused by cancer drugs. An even smaller proportion (12.4%) remembered that their doctor informed them about possible cardiac complications caused by chest radiotherapy. Nearly 32% of patients were not examined by a cardiologist during cancer treatment, and more than half (53.5%) underwent echocardiography during cancer treatment. However, 95.5% of patients believed that every patient undergoing cancer treatment should have a cardiology consultation.

Patients who were informed about possible complications and underwent cardiological examinations had better knowledge about complications and the need to monitor their heart after cancer treatment.

### 3.4. Health-Promoting Behaviors in Cardio-Oncology Patients

Table 4 presents results from Health Behavior Scale domains. An overview of the specific questions is presented in the Supplementary Material (Table S1). Out of a possible 45 points, respondents scored an average of 24.21 (SD = 8.18) points. Preventive behaviors related to the healthcare system received the lowest score (1.33 out of 3 possible), which indicates that patients diagnosed with cancer relatively rarely underwent preventive prostate cancer screening or smear test. On the other hand, patients scored highest in the Unhealthy behaviors domain, which indicates that they rarely consume tobacco or alcohol products (2.05 ± 0.46).

The Mann–Whitney U test indicated a statistically significant association between patients’ health behaviors and cardio-oncology knowledge (*p* < 0.001). Individuals who knew what cardio-oncology is and provided correct answer declared healthier behaviors (M = 27.9 ± 6.0) than those who did not have this knowledge (M = 23.1 ± 8.5). Patients who were aware that chemotherapy and radiotherapy could have negative cardiac consequences also exhibited better health behaviors related to diet, physical activity or healthcare system (Table 5).

## 4. Discussion

### 4.1. Complications Associated with Chemotherapy and Radiation Therapy

Among those who completed our questionnaire, 54 participants (22.22%) reported receiving chemotherapy, while 79 (32.51%) received both chemotherapy and radiation therapy. Radiation therapy alone was used in 45 individuals (18.52%). Notably, 65 participants (26.75%) did not know what type of cancer treatment they had undergone.

It is concerning that such a large group of patients, despite having undergone potentially cardiotoxic therapy, were not aware of the need for regular follow-up. Nearly half of them had received chest radiotherapy, and it is well known that its complications increase over time—coronary artery disease or valvular dysfunction may appear even 20 years after treatment. In the absence of follow-up, patients often present to a cardiologist only at an advanced stage of disease. Even more striking are the data showing that more than 26% of patients in the cardio-oncology clinic did not know what type of oncologic therapy they had received. It is difficult to determine whether this results from denial of the cancer diagnosis, lack of interest in their health despite the disease, or concomitant depression.

Nutbeam et al. already in 2000 defined health literacy (HL) as a set of skills and knowledge that enables individuals to interpret and use health-related information properly [20]. This ability appears to be crucial for cancer patients in making decisions at every stage of treatment. Researchers have identified limited HL in so-called socially vulnerable populations, such as oncology patients. Similarly, to our study population, lower levels of knowledge have also been reported among oncology groups. As demonstrated by other researchers, this is associated with an increased number of hospitalizations, more frequent use of emergency care, and poorer overall health status. In oncology patients, limited HL is linked to lower participation rates in screening programs and a more advanced stage of cancer at diagnosis [21].

The patient’s active role in cardio-oncologic follow-up is essential for the effective detection and prevention of cardiovascular complications. Patients should be aware of the cardiotoxic risks associated with cancer therapy and recognize symptoms that warrant medical attention.

This is especially relevant for late complications of radiation therapy. A patient may attempt to move on from their cancer diagnosis, but they must remain aware of the potential cardiovascular risks that may still emerge years later [22].

### 4.2. Knowledge About Cardiac Complications

Patients undergoing oncology treatment are very often unaware of the link between oncological treatment and cardiac complications. Oncology patients still have limited knowledge about cardiovascular complications associated with cancer treatment [23]. In our study, the questionnaire was completed by patients of the cardio-oncology outpatient clinic operating within the Department of Cardiology. The existing knowledge gap lies in the fact that patients are not aware of the need for follow-up after treatment, were not informed about it, or were informed ineffectively since they do not recall the information provided, and some even assumed that no follow-up was necessary. In the literature, there are virtually no studies systematically assessing patients’ awareness in this area, which highlights the importance of our findings and justifies that this study makes a significant contribution to the existing body of knowledge, pointing to the urgent need for educational efforts. In our survey, 72 people (29.6%) were not concerned about cardiac complications. As many as 66.3% believed that after chemotherapy, there was no need to monitor the heart attempt [23]. The data on thoracic radiotherapy are even more disturbing: only 32.1% of patients thought it was harmful to the heart. Furthermore, only 20.2% of patients believed that these complications could occur even 10 years after the end of radiation therapy. Although not every patient takes an active interest in their treatment, it is surprising that in this group of respondents, as many as 46.9% rated their health knowledge as very good or good [23,24]. Although our study group consisted of patients who voluntarily attended follow-up visits at a cardio-oncology clinic, this suggests they should be more aware of the need for such monitoring. Their level of knowledge about potential complications turned out to be surprisingly low. Moreover, many of them rated their own health knowledge as good or very good. This raises the question of what the level of awareness is among oncology patients who do not attend. As shown in other studies, individuals with higher education usually possess greater knowledge about their health status. A higher level of education is also associated with better health literacy, which translates into easier understanding of medical information and more active participation in the treatment process [25]. Better-educated patients are more likely to use modern sources of information, such as the Internet, and to ask their physicians questions. In addition, they are more likely to engage in preventive behaviors and attend follow-up examinations, which facilitates earlier disease detection and improves prognosis [26]. Is their knowledge so limited that it leads to a complete lack of specialist consultations? White et al. report that the majority of patients in an Australian clinic—15 people aged 38–74 (10 women, 5 men)—did not know that their cancer treatment could increase their risk of cardiovascular disease [27]. More often than not, it was women with breast cancer treated with trastuzumab who reported that they had been informed of the risk of CVD, and this awareness was reinforced by regular ECG monitoring. On the other hand, however, a lack of knowledge and the ability to recognize the symptoms of cardiovascular disease can result in a late diagnosis of cardiovascular complications. White et al. emphasize that patients openly expressed how difficult it was to cope with the demands of managing two chronic diseases—cancer and cardiovascular disease [27].

Among our respondents, those with a college education were more knowledgeable about cardio-oncology. Of the 92 people with a college education, 34 people (36.96%) were aware of what cardio-oncology does. The situation is similar for overweight and obese patients. Among this group of patients, 19 people (29.92%) knew what cardio-oncology does. Of this relatively young branch of medicine, 43.75% of those in the 18–40 age bracket were aware, and 25.41% of those in the 41–54 age bracket. People over 55 knew the least about cardio-oncology (12.7% of those in the 55–64th year bracket and 15.38% of those over 65). Older people often have a lower level of knowledge about cardiac complications and cardio-oncology due to limited access to modern sources of information, such as the Internet. They often rely solely on word-of-mouth communication from their doctor. This message can often be concise, difficult to remember, and many times, in the case of cardio-oncology, medical personnel do not educate oncology patients at all. Hearing, memory, or concentration problems can also be an issue in this older population. These can further hinder the assimilation of medical information. In addition, older people are less likely to ask questions of the doctor, assuming that they have no say in the treatment plan [28].

In their review, Waddell et al. also pointed out that a significant majority of respondents admitted that they were not at all aware of potential cardiac complications following cancer treatment. Patients also admitted that they were not informed of this fact by anyone on the medical staff [29]. Only a small number of patients had this knowledge, and it was communicated to them by their oncologist. The researchers also point out that perhaps the diagnosis of oncologic disease is so overwhelming to patients that it overshadows potential cardiovascular disease. This fact is important because most of the participants in this study already had cardiovascular disease (CVD) risk factors present at the onset of their cancer disease. Since cancer and heart disease share many common risk factors, people who start oncology treatment with risk factors already present at the start are at high risk of developing cardiovascular complications.

The patient’s active role in cardio-oncologic follow-up is essential for the effective detection and prevention of cardiovascular complications. Patients should be aware of the cardiotoxic risks associated with cancer therapy and recognize symptoms that warrant medical attention. This is especially relevant for late complications of radiation therapy [22].

### 4.3. The Physician’s Role in Communicating Cardiac Complications

A survey of cardiologists, oncologists, and general practitioners revealed that 83% believe oncologists should be mindful of cardiovascular complications in their patients. However, the study found that only half of oncologists discuss cardiotoxicity with their patients, highlighting a significant communication gap [30].

Clark et al. analyzed 50 cases of confirmed cardiotoxicity. In this group, up to 89% of patients already had CVD risk factors before chemotherapy [31]. Despite this, only 15% were referred to a cardiologist before starting treatment. Even after the onset of cardiotoxicity, just 57% were referred to a cardiology clinic. Following the completion of cancer therapy, 48% were referred to heart failure clinics, 17% to cardiac rehabilitation, and only 2% to oncology outpatient clinics. The study concluded that oncologists rarely informed patients about the risk of cardiotoxicity, even among those at high risk. The authors emphasized the need to create structured pathways that integrate cardiology and oncology, targeting both patient and provider education [32].

In another study, the majority of participants reported being referred to a cardio-oncology clinic early in their cancer treatment. Referrals were based on pre-existing cardiovascular conditions or the emergence of concerning symptoms. Patients noted that the inclusion of cardiac care made them feel more secure and provided psychological reassurance. Importantly, the referral did not increase anxiety about heart disease. On the contrary, early cardiac follow-up gave them hope that any complications would be detected and managed promptly [27].

However, the same group of patients reported receiving very limited information from oncologists about modifying cardiovascular risk factors. They believed that the responsibility to provide this information should lie with nurses, oncologists, or primary care physicians, and that such education should occur at the beginning of cancer treatment so that patients could respond appropriately to any symptoms.

Some participants in another study emphasized that the timing of cardiovascular risk assessment and treatment initiation played an important role in determining their engagement with cardiac care. While preferences varied, some patients expressed that it would be more comfortable to focus on cardiac risk only after completing cancer therapy [31].

Among our patients, as many as 134 individuals (55.1%) reported that they had not received any information from their oncologist regarding potential complications after chemotherapy. Fifty patients (20.58%) could not recall whether such information had been provided, while only 59 patients (24.28%) confirmed that they had been informed about the risk of cardiotoxicity.

When asked whether cardiac monitoring is necessary after chemotherapy, the vast majority—80.65%—answered affirmatively, 16.04% were unsure, and 3.2% believed there was no need for it. However, the responses differed significantly when the question pertained to the need for cardiac monitoring after radiation therapy (32.9% of respondents answered affirmatively) [23].

When asked whether they had been informed by their physician about possible complications following radiation therapy, the responses were as follows: 139 individuals (57.20%) stated they had not been informed, 74 (30.45%) did not remember, and only 30 (12.35%) confirmed receiving such information.

In contrast, patients who recalled receiving information about potential cardiac complications associated with oncology treatment demonstrated more favorable health-related attitudes. Similarly, those who remembered undergoing an echocardiogram during their oncology treatment were more likely to engage in health-promoting behaviors. This association may also be related to patients’ health literacy. According to the definition of health literacy, people with higher levels of health literacy are better at remembering the health information needed to make health-related decisions [33]. Accordingly, health education delivered by a doctor in their office would bring substantial benefits.

Health education provided by an oncologist or cardiologist plays a crucial role in raising patient awareness about potential complications of cancer therapy. The lack of awareness among oncology patients regarding the need for cardiology follow-up after treatment leads to delayed recognition of complications and worse prognosis. This lack of knowledge may result from the fact that patients either did not receive such information from their physician, or it was delivered in such an unclear way that they did not remember it. As a consequence, patients fail to undertake preventive actions, and complications are recognized late. Patients who receive clear and comprehensible information are more likely to monitor their health regularly. Conversely, a lack of education or low health literacy may result in the neglect of symptoms and delays in diagnosis. Therefore, health education should be an integral component of care for oncology and cardio-oncology patients [34].

### 4.4. Health-Promoting Behaviors in Cardio-Oncology Patients

In their publication, White and co-authors noted that for some patients, a diagnosis of cardiovascular disease during oncology treatment served as a motivation to change their lifestyle. However, the majority of individuals in the study struggled to implement health-related modifications. This finding is not surprising, as previous research indicates that people with comorbidities often face difficulties in establishing health priorities. Their focus frequently remains on “living in the moment” and coping with the immediate challenges of survival [27]. The study also demonstrated that early access to cardio-oncology services improved patients’ understanding of the link between cancer therapy and cardiotoxicity.

In our study, patients who were aware of the need for cardiac monitoring following radiation therapy and chemotherapy scored higher in health behaviors. These individuals were more likely to undergo preventive screenings, physical exercises, and a healthier diet.

Lifestyle plays a crucial role at every stage of the cancer journey—it influences cancer risk, treatment outcomes, rehabilitation, and the development of cardiovascular complications [35]. Similar to cardiovascular diseases, physical activity and a healthy diet significantly improve survival rates after cancer.

Patients who have undergone treatment for cancers such as testicular, breast, or colorectal cancer are at greater risk of developing cardiovascular disease compared to the general population. This is primarily due to the cardiotoxic effects of certain therapies. Chemotherapy (e.g., anthracyclines, cisplatin) and radiation therapy targeting the thoracic or pelvic regions can cause lasting damage to the heart and blood vessels. Furthermore, cancer treatments often lead to metabolic disturbances—including weight gain, hypertension, and lipid disorders—which are recognized risk factors for cardiovascular disease [32,35].

Our analysis revealed findings consistent with those of the Korea National Health and Nutrition Examination Survey (2013–2021), which included 2597 cancer survivors and 2458 individuals without a cancer history. In both analyses, survivors who have more than five years post-diagnosis were more likely to attend general health check-ups but less likely to undergo recommended cancer screenings. They also demonstrated less consistency in managing chronic conditions, while reporting lower rates of smoking and alcohol use [36]. Moreover, our findings on preventive behaviors among cancer patients indicate that the mean HBS scores across domains reflect a predominance of unfavorable health behaviors. Compared with the previously studied population of primary care patients, participants of this study exhibited a markedly poorer behavioral profile [37].

The 2022 ESC guidelines emphasize that effective physician–patient communication is a key element in the prevention and early detection of cardiovascular complications following cancer treatment. Risk information should be delivered clearly, reinforced at subsequent visits, and tailored to the patient’s individual cognitive abilities. Such a communication model increases patients’ awareness, their engagement in the therapeutic process, and promotes earlier reporting of concerning symptoms. The guidelines also highlight that an organized cardio-oncology clinic plays a crucial role in raising awareness of cardiotoxicity among oncology patients. A structured approach to cardio-oncology units and outpatient clinics, such as ours, enhances patients’ understanding and adherence to recommendations while also facilitating early recognition of cardiotoxicity, ultimately leading to better clinical outcomes [1]. Risk stratification in oncology patients is a key element of cardio-oncology therapy. As emphasized in the 2022 ESC guidelines, the HFA-ICOS risk score allows for the assessment of individual cardiotoxicity risk depending on the type of therapy, comorbidities, and demographic factors. Incorporating this score into clinical practice enables better tailoring of monitoring and the implementation of preventive measures in high-risk patients. Importantly, dedicated cardio-oncology teams should be responsible for performing this risk assessment before the initiation of oncological therapy [1].

The education of oncology patients should be a routine, systematic, and repeated part of every oncology and cardiology visit. Clear and repeated communication of cardiovascular risk and promotion of healthy behaviors is essential, ideally supported by written or multimedia materials. Educational programs should involve both patients and their families to strengthen the message and enhance its effectiveness. It is also important to create care pathways in which the oncologist and cardiologist jointly monitor the patient, ensuring consistent and understandable recommendations.

## 5. Conclusions

Cardio-oncology is becoming an indispensable component of comprehensive care for oncology patients. As cancer treatments continue to improve in effectiveness, they are increasingly associated with a risk of cardiovascular complications that can significantly impair patients’ quality of life and affect overall prognosis. The early involvement of a cardiologist at the stage of planning oncological therapy allows for individualized risk assessment and timely implementation of preventive measures.

Unfortunately, as demonstrated by our study, the level of knowledge among oncology patients regarding potential complications of chemotherapy and radiotherapy remains insufficient. There is a clear need to implement effective educational programs aimed at increasing awareness of these risks and fostering stronger interdisciplinary collaboration between oncologists, cardiologists, and primary care physicians.

Greater emphasis should be placed on prevention through the control of comorbidities that already increase baseline cardiovascular risk, such as hypertension, diabetes, and obesity, even before the initiation of cancer treatment. Regular cardiac monitoring during and after therapy should become the standard of care. Patients must also receive clear and comprehensible information about the potential adverse cardiovascular effects of oncological treatment.

Improved patient education may enhance patient engagement in the treatment process and facilitate the early detection of cardiac complications. As shown in our survey, patients who were informed by their physician about the potential cardiotoxicity of oncological therapies demonstrated more health-conscious behaviors. Early identification of cardiotoxicity enables timely intervention, reducing the likelihood of long-term complications. This proactive approach not only enhances treatment safety but also strengthens patients’ trust in the healthcare system as a whole.

## 6. Limitations

The following study had a few limitations. Considering that the research sample consisted of a conveniently selected group of patients, the risk of selection bias had to be taken into account. This method of participant recruitment may result in limited diversity in the study population. On the other hand, focusing on a specific group of oncology patients allowed for an in-depth analysis of the issue in a clinical context. It increases the practical value of the results for health professionals working with a similar patient profile.

In addition, the study was single-center and was conducted exclusively in one cardiology clinic, which also limits the representativeness of the sample. The organizational conditions and the patient profile of this particular facility may differ from other centers. However, the results obtained can serve as a starting point for developing measures to improve the care of oncology patients in similar facilities.

Data collection was carried out using self-reported questionnaires, which are associated with a number of possible methodological limitations. The subjectivity may affect the accuracy of the responses. On the other hand, patients filling out the questionnaires on their own might have encouraged them to be more open and honest in their answers, which could be challenging in face-to-face interviews.

## Figures and Tables

**Table 1:** Sample characteristics.

*n* = 243		*n*	%
**Sex**	Female	184	75.7
	Male	59	24.3
**Age**	18–40	32	13.2
	41–54	122	50.2
	55–64	63	25.9
	65+	26	10.7
**Education**	Primary (+vocational)	57	23.5
	Secondary	94	38.7
	Higher	92	37.9
**Residence ^1^**	Village	15	6.2
	Village in an urban agglomeration	39	16.1
	Small/medium city	59	24.3
	Large city	130	53.5
**Occupational status**	I work actively	150	61.7
	Other ^2^	93	38.3
**Financial situation**	Very bad	57	23.5
	Bad	74	30.5
	Average	90	37
	Good/Very good	22	9.1
**BMI**	Normal	113	46.5
	Overweight	98	40.3
	Obesity	32	13.2
**Cancer type**	Ovarian	24	9.9
	Breast	135	55.6
	Lymphoma	41	16.9
	Other ^3^	42	17.3
**Therapy type**	Chemotherapy ^4^	54	22.2
	Radiotherapy	45	18.5
	Chemotherapy + radiotherapy	79	32.5
	I don’t know	65	26.8

^1^ small city: up to 20,000 inhabitants, medium city: 20,000–100,000 inhabitants, large city: above 100,000 inhabitants [7]; ^2^ currently unemployed, rehabilitation benefit, sick leave, maternity/parental leave, pension; ^3^ lung cancer, kidney cancer, leukemia, uterine cancer; ^4^ including doxorubicin, cyclophosphamide and immunotherapy (nivolumab).

**Table 2:** Associations between cardio-oncology knowledge and sociodemographic data.

	Cardio- Oncology Knowledge	Chemotherapy Can “Damage” the Heart	The Heart Should Be Monitored During Chemotherapy	One Should Go to a Cardiologist for Follow-Up After Cancer Treatment	The Heart Should Be Monitored After Radiation Therapy to the Chest	Radiation Therapy to the Chest Can “Damage” the Heart
	CORRECT	YES	YES	YES	YES	YES
**Age**						
18–40 (*n* = 32)	14 (43.8%)	30 (93.75%)	31 (96.88%)	25 (78.13%)	21 (65.63%)	16 (50%)
41–54 (*n* = 122)	31 (25.4%)	98 (80.33%)	106 (86.89%)	87 (71.31%)	49 (40.16%)	49 (40.16%)
55–64 (*n* = 63)	8 (12.7%)	39 (61.9%)	41 (65.08%)	36 (57.14%)	8 (12.7%)	9 (14.29%)
65+ (*n* = 26)	4 (154%)	15 (57.69%)	18 (69.23%)	13 (50%)	2 (7.69%)	4 (15.38%)
*p* value	**0.001**	**<0.001**	**<0.001**	**0.003**	**<0.001**	**<0.001**
**Education**						
primary/vocational (*n* = 57)	5 (8.8%)	29 (50.88%)	31 (54.39%)	27 (47.37%)	4 (7.02%)	6 (10.53%)
secondary (*n* = 94)	18 (19.2%)	70 (74.47%)	78 (82.98%)	56 (59.57%)	20 (21.28%)	19 (20.21%)
higher (*n* = 92)	34 (37.0%)	83 (90.22%)	87 (94.57%)	78 (84.78%)	56 (60.87%)	53 (57.61%)
*p* value	**<0.001**	**<0.001**	**<0.001**	**<0.001**	**<0.001**	**<0.001**
**Financial situation**						
very bad (*n* = 57)	21 (36.8%)	51 (89.47%)	53 (92.98%)	47 (82.46%)	25 (43.86%)	27 (47.37%)
bad (*n* = 74)	12 (16.2%)	57 (77.03%)	62 (83.78%)	51 (68.92%)	18 (24.32%)	18 (24.32%)
average (*n* = 90)	20 (22.2%)	57 (63.33%)	63 (70%)	48 (53.33%)	25 (27.78%)	22 (24.44%)
good/very good (*n* = 22)	4 (18.2%)	17 (77.27%)	18 (81.82%)	15 (68.18%)	12 (54.55%)	11 (50%)
*p* value	0.081	**0.003**	**0.003**	**0.002**	0.789	0.225
**BMI**						
normal (*n* = 113)	38 (33.6%)	90 (79.65%)	96 (84.96%)	79 (69.91%)	47 (41.59%)	42 (37.17%)
overweight (*n* = 98)	14 (14.3%)	67 (68.37%)	75 (76.53%)	58 (59.18%)	19 (19.39%)	21 (21.43%)
obesity (*n* = 32)	5 (15.6%)	25 (78.13%)	25 (78.13%)	24 (75%)	14 (43.75%)	15 (46.88%)
*p* value	**0.001**	0.250	0.153	0.592	0.085	0.525
**Therapy type**						
chemotherapy (*n* = 54)	14 (25.9%)	45 (83.33%)	52 (96.3%)	37 (68.52%)	23 (42.59%)	20 (37.04%)
Radiotherapy (*n* = 45)	10 (22.2%)	28 (62.22%)	32 (71.11%)	32 (71.11%)	12 (26.67%)	13 (28.89%)
I don’t know (*n* = 64)	6 (9.4%)	38 (59.38%)	42 (65.63%)	33 (51.56%)	8 (12.5%)	8 (12.5%)
chemotherapy + radiotherapy (*n* = 79)	27 (34.2%)	70 (88.61%)	69 (87.34%)	58 (73.42%)	37 (46.84%)	37 (46.84%)
*p* value	**0.006**	**<0.001**	**<0.001**	**0.036**	**<0.001**	**<0.001**

**Table 3:** Associations between knowledge and doctors’ actions.

	Heart should Be Monitored During Chemotherapy	One Should Go to a Cardiologist for Follow-Up After Cancer Treatment	Radiation Therapy to the Chest Can “Damage” the Heart
	yes	yes	yes
The doctor informed about possible cardiac complications caused by cancer drugs			
yes	58 (98.3%)	47 (79.7%)	29 (49.2%)
no/I don’t remember	138 (75.0%)	114 (62.0%)	49 (26.6%)
*p* value	**<0.001**	**0.012**	**0.001**
The doctor informed about possible cardiac complications caused by radiation therapy to the chest			
yes	29 (96.7%)	26 (86.7%)	21 (70.0%)
no/I don’t remember	167 (78.4%)	135 (63.4%)	57 (26.8%)
*p* value	**0.018**	**0.012**	**<0.001**
cardiologist examined one’s during cancer treatment			
yes	142 (85.5%)	121 (72.9%)	55 (33.1%)
no/I don’t remember	54 (70.1%)	40 (51.9%)	23 (29.9%)
*p* value	**0.005**	**0.001**	0.612
Echocardiogram during one’s cancer treatment			
yes	115 (88.5%)	104 (80.0%	56 (43.1%)
no/I don’t remember	81 (71.7%)	57 (50.4%)	22 (19.5%)
*p* value	**0.001**	**<0.001**	**<0.001**

**Table 4:** Results of HBS domains.

	M ^1^	SD ^2^
Total score HBS	24.21	8.18
D1: Preventive behaviors related to healthcare system	1.33	0.81
D2: Individual preventive behaviors	1.59	0.58
D3: Health behaviors related to diet	1.72	0.87
D4: Health behaviors related to physical activity	1.53	0.97
D5: Unhealthy behaviors	2.05	0.46

^1^ mean; ^2^ standard deviation.

**Table 5:** Associations between knowledge and health behaviors.

Cardio-Oncology Knowledge	Incorrect	Correct	*p* Value
	23.07 ± 8.47	27.89 ± 5.96	**<0.001**
	**no/I don’t remember**	**yes**	***p*** **value**
chemotherapy can “damage” the heart	20.05 ± 9.46	25.61 ± 7.21	**<0.001**
Heart should be monitored during chemotherapy	18.09 ± 8.5	25.68 ± 7.4	**<0.001**
One should go to a cardiologist for follow-up after cancer treatment	20.59 ± 8.43	26.06 ± 7.43	**<0.001**

## Data Availability

The anonymized data presented in this study are available on request from the corresponding author.

## Supplementary Materials

The following supporting information can be downloaded at https://www.mdpi.com/article/10.3390/curroncol32110613/s1, Table S1: Health Behaviour Scale.
