# Supplementary material for: The Cardio-Oncology Patients—What They Know and What They Should Know
Source: Curr Oncol. 2025 Nov 2;32(11):613. doi: 10.3390/curroncol32110613 (PMC12651008; doi:10.3390/curroncol32110613)
Supplement: Supplementary file 1 [file curroncol-32-00613-s001.zip › curroncol-3802700-supplementary.pdf]

The Cardio-oncology Patient – What They Know and What They Should Know?

Supplementary Material

Table S1. Health Behaviour Scale

D1: Preventive behaviours related to healthcare system				
I perform a smear test (only for women)	**once every 6 months**	**once a year**	**once every few years**	**never**
I have a medical examination for prostate cancer (only for men)	more often than once a year	once a year	once every few years	never
I go to the dentist for preventive check-ups	**once every 6 months**	**once a year**	**once every few years**	**never**
I check my blood pressure	**every day**	**several times a week**	**several times a month**	**several times a year or never**
D2: Individual preventive behaviours				
I control my body weight	**once a month or more often**	**once or several times a year**	**once every few years**	**never**
I check my body for physical lesions or abnormalities	**several times a month**	**once a month**	**once or several times a year**	**never**
I can effectively manage stress	**yes, always**	**yes, sometimes**	**yes, rarely**	**never**
D3: Health behaviours related to diet				
My diet is varied	**yes**	**rather yes**	**rather no**	**no**
I limit the consumption of sugar and foods which contain it (sweets)	**yes**	**rather yes**	**rather no**	**no**
When buying food products. I check their composition	**yes**	**rather yes**	**rather no**	**no**
I eat 4-5 portions of fruit and vegetables per day (1 portion is one fruit or one vegetable)	**yes**	**rather yes**	**rather no**	**no**
D4: Health behaviours related to physical activity				
I lead an active lifestyle	**yes**	**rather yes**	**rather no**	**no**
I use daily activities as an opportunity for physical activity (e.g.. I climb the stairs instead of using the elevator. park my car at a distance so that I can walk. I move around by bicycle)	**yes**	**rather yes**	**rather no**	**no**
I regularly perform physical exercise	**at least 3 times a week**	**1-2 times a week**	**several times a month**	**never**
D5: Unhealthy behaviours				
I consume alcohol drinks	**every day**	**several times a week**	**several times a month**	**several times a year or never**
I smoke cigarettes	**every day >5**	**every day <5**	**not anymore**	**never**
